# Oblique lateral interbody fusion in heterogenous lumbar diseases: Anterolateral screw fixation vs. posterior percutaneous pedicle screw fixation – A single center experience

**DOI:** 10.3389/fsurg.2022.989372

**Published:** 2022-12-26

**Authors:** Meng-Ting Wu, Tzu-Tsao Chung, Shao-Ching Chen, Tzu-Jen Kao, Wen-Shin Song

**Affiliations:** ^1^ Division of Neurosurgery, Department of Surgery, Cheng-Hsin General Hospital, Taipei, Taiwan; ^2^ Ph.D. Program of Electrical and Communications Engineering, Feng Chia University, Taichung City, Taiwan; ^3^ Department of Neurological Surgery, Tri-Service General Hospital, National Defense Medical Center, Taipei, Taiwan; ^4^ Institute of Neuroscience, National Yang Ming Chiao Tung University, Taipei, Taiwan; ^5^ Ph.D. Program in Medical Neuroscience, Taipei Medical University, Taipei, Taiwan; ^6^ International Master Program in Medical Neuroscience, Taipei Medical University, Taipei, Taiwan; ^7^ Department of Surgery, Tri-Service General Hospital, National Defense Medical Center, Taipei, Taiwan

**Keywords:** anterolateral screw fixation, posterior percutaneous screw fixation, heterogenous lumbar disease, lumbar fusion, oblique lateral interbody fusion, navigation

## Abstract

**Background:**

Oblique lateral interbody fusion (OLIF) is a type of minimally invasive lateral lumbar interbody fusion technique used for treating lumbar degenerative diseases. This study aimed to analyze the clinical and radiographic efficacy of OLIF with anterolateral screw fixation alone and OLIF requiring fixation with conventional posterior percutaneous pedicle screws for lumbar diseases.

**Methods:**

Medical records of consecutive patients admitted to Cheng-Hsin Hospital who received OLIF between January 2019 and December 2020 were retrospectively reviewed. Patients were divided into two groups by screw fixation: patients who received anterolateral screw fixation alone were defined as one-stage OLIF (*n* = 9) and patients who received fixation with conventional posterior percutaneous pedicle screw were defined as two-stage OLIF (*n* = 16). Patient clinical characteristics, medical history, intraoperative blood loss, length of hospital stay, peri-operative, and post-operative complications were evaluated in all patients.

**Results:**

During the study period, a total of 25 patients were successfully treated with OLIF (*n* = 9 one-stage; *n* = 16 two-stage). Two-stage OLIF was associated with longer operation times, longer hospital stays, shorter bed-rest time, and a greater likelihood of having a blood transfusion compared with the one-stage OLIF group. A higher proportion of grade I subsidence was observed at 6 months and 1 year after surgery in the two-stage group compared with the one-stage group. Post-operative complications included ileus, dystonia, and dystonia were higher in the two-stage OLIF group. Improvements in radiographic parameters were demonstrated after OLIF, and the improvements were comparable between one-stage and two-stage OLIF.

**Conclusions:**

One-stage OLIF is a feasible and efficacious treatment method for single- and multiple-level degenerative lumbar diseases. Additional clinical follow-up is necessary to confirm long-term outcomes.

## Introduction

Low back pain (LBP) is the most common musculoskeletal problem globally (1–4) and results in significant medical burden and economic cost (2, 5). If patients are not responding to conservative treatments and their quality of life is affected due to LBP and lumbar diseases such as degenerative disc disease, disc herniations, stenosis, spondylolisthesis, and scoliosis surgery may be required. Oblique Lateral Interbody Fusion (OLIF) is a type of minimally invasive lateral lumbar interbody fusion technique with a route of insertion at the psoas major used for indirect decompression of the neural structures through interbody distraction and fusion in the lumbar spine (6, 7). It utilizes a similar approach as anterior lumbar interbody fusion (ALIF) but is also considered a lateral lumbar interbody fusion (LLIF). When comparing the surgical outcome of OLIF, LLF, and ALIF, the anterior approach remains the most favorable technique for wide fusion, indirect decompression, and segmental lordosis reconstruction, however, the anterior approach is also heavily dependent on the surgeon's skill and experience in handling the visceral and vascular structures. OLIF has several noteworthy advantages, including less operative bleeding, a lower rate of nerve injury, and faster recovery compared with traditional posterior surgery (6, 8). A meta-analysis of 56 studies compared the radiographic and clinical outcomes of OLIF and LLIF for degenerative lumbar disease concluded that the two approaches are similar in terms of radiographic outcomes, the incidence of perioperative complications, operative blood loss, operative time, and the length of hospital stay. Greater improvement in VAS and ODI scores was found with the OLIF approach. Importantly, the incidence of main complications is significantly different, a higher rate of nerve injury and psoas weakness were found in LLIF, while the complications associated with OLIF were minor vascular injury (9) and cage subsidence (10), therefore supplemental posterior percutaneous pedicle screw fixation following OLIF is often recommended to improve spinal stability and intervertebral fusion rates (11, 12). Currently, after lateral access surgery, the patient is repositioned in the prone position for pedicle screw fixation which requires a second round of surgery preparation, which significantly increases operation time, risk of complication, cost, and radiation exposure (13). An alternative approach is anterolateral screw fixation (14). Due to the larger OLIF cage and the stability of the fixation interface, fixation can be achieved with anterolateral screw-assisted fixation of the vertebral body requiring no change of patient position (15, 16).

No clear guidance or clinical data exists regarding the choice of fixation approach with OLIF, specifically whether to use a one-stage or two-stage surgical approach. Furthermore, most studies on this topic reported outcomes for single-level lumbar disease. The current study examined the surgical approach to treating lumbar diseases according to the position of supplemental percutaneous pedicle screw fixation following OLIF, including in patients with multiple-level and varied medical histories and complications.

## Methods

### Patients

This is a retrospective observational study of patients who underwent OLIF at our institution. The medical records of patients admitted to Cheng-Hsin Hospital who received OLIF between January 2019 and December 2020 were retrospectively reviewed. The inclusion criteria were as follows: (1) age >18 years, (2) patients who received OLIF, and 3) follow-up duration >12 months. Patients were grouped into those who received one-stage OLIF or two-stage OLIF. One-stage OLIF was defined by having anterolateral screw fixation alone and two-stage OLIF was defined by having conventional posterior percutaneous pedicle screws. Patients who received one stage OLIF include: (1) those without spinal stenosis or only have mild spinal stenosis; (2) adjacent segment disease who had previous TLIF or. PLIF. Patient clinical characteristics, medical history, intraoperative blood loss, length of hospital stay, and peri-operative and post-operative complications were collected. This study was approved by the Ethics committee of Cheng-Hsin Hospital [IRB number: (857) 110-3] and was conducted in accordance with the Declaration of Helsinki and reported in line with the STROBE criteria. Written informed consent was obtained from all patients.

### Surgical procedure

The same experienced clinician performed the surgery for all patients. The surgery was performed in line with the methods reported by Sato et al. (17) and Guo et al. 2022 (18). Briefly, the patient underwent general anesthesia and was placed in the right decubitus position, and the target segment was identified using C-arm fluoroscopy. The abdominal muscles were bluntly dissected ventrally from the target intervertebral space to the retroperitoneal space. The fingers pushed the peritoneum forward to the anterior edge of the psoas major and ventrally to expose the space between the psoas major and the abdominal aorta. The target intervertebral disc was touched, and a needle and the C-arm were positioned. After the target intervertebral disc was confirmed, the OLIF tubular retractor system was placed. Lastly, the expanding trocar was connected to the serpent arm and fixed on the operating table. Stabilizing pins were placed at the lower endplate level of the upper vertebral body to secure the working channel, which was moderately extended to fully expose the target disc. Residual disc tissue was treated with a reamer and the endplate cartilage was processed. The mold of the cage was tested and examined under a fluoroscope. A suitable cage mixed with allograft bone was loaded into the target intervertebral space and positioned through fluorosco­py.

### Radiographic evaluation

Imaging data were collected before surgery, 1-week, 3-, 6-, and 12 months after surgery, as well as at the final follow-up visit. Fusion rates were evaluated at 12 months post-surgery, and grading was defined based on the Bridwell interbody fusion scoring system from grade I to IV (grades I and II are indicative of successful fusions) (19).

Subsidence was evaluated at 3-, 6- and 12 months after surgery. Subsidence grade was defined according to Sharma et al. (2011) (20) from grade 0 to grade III. Grade 0 represents a normal end plate without fracture; Grade I represent a breach of the end plate at one side (anterior or posterior) of the cage; Grade II represents a fracture of the end plate at both the anterior and posterior sides of the cage, and Grade III represents an end-plate fracture with cage subsidence of more than one-third of the cage height into the vertebral body.

Changes in anterior disc height (ADH), posterior disc height (PDH), foraminal height (FH), foraminal width (FW), retrolisthesis index (RI), angle of L1-S1, angle of disc level coronal/sagittal, lumbar lordosis (LL), between pre- and post-op were evaluated using x-ray imaging. All images were examined by two independent physicians with >10 years of experience blinded to each other's results, the interobserver reliability for all measurements was good to excellent as determined using the intraclass correlation coefficient.

### Statistical methods

Continuous variables are presented as mean and standard deviation (SD). Categorical variables are presented as counts and percentages. Shapiro-Wilk test was used to test the normality for continuous variables. Wilcoxon sign rank test was used to evaluate radiographic parameters before and after surgery. A t-test was used to evaluate differences between groups over time. Intraclass correlation coefficients were used to assess interobserver reliability. Results were considered significant at *p* < 0.05. All statistical analyses were performed using SAS version 9.4, Windows NT version (SAS Institute, Inc., Cary, NC, United States).

## Results

A total of 25 patients underwent the OLIF surgical procedure and were included in the study population. Nine patients underwent the one-stage procedure and 16 underwent the two-stage procedure. Patient baseline characteristics are presented in Table 1. Overall, patients were similar in age (mean: 68.1 years). The majority of patients who underwent the two-stage surgery, compared with those who underwent the one-stage procedure, were male (31.3% vs. 22.2%), had hypertension (50% vs. 33.3%), stenosis (6.3% vs. 0%), spondylolisthesis (81.3% vs. 77.8%), degenerative disc disease (12.5% vs. 0%), no history of spine surgery (93.8% vs. 66.7%), osteoporosis (43.7% vs. 33.3%), BDM ≤ −3.0 (31.3% vs. 11.1%), treated for 2 levels (25% vs. 11.1%), L4-5 surgery (68.8% vs. 33.3%), and posterior decompression (62.5% vs. 11.1%).

**Table 1:** Baseline and clinical data.

	Total, *n* = 25	One-stage, *n* = 9	Two-stage, *n* = 16
Age (year)	68.1 ± 8.8	67.8 ± 9.8	68.3 ± 8.6
Sex			
Male	7 (28.0)	2 (22.2)	5 (31.3)
Female	18 (72.0)	7 (77.8)	11 (68.8)
Hypertension	11 (44.0)	3 (33.3)	8 (50.0)
Hyperlipidemia	6 (24.0)	4 (44.4)	2 (12.5)
DM	5 (20.0)	3 (33.3)	2 (12.5)
Chronic renal disease	1 (4.0)	1 (11.1)	0
Diagnosis (*n*, %)			
Stenosis	1 (4.0)	0	1 (6.3)
Spondylolisthesis	20 (80.0)	7 (77.8)	13 (81.3)
Degenerative disc disease	2 (8.0)	0	2 (12.5)
Rupture disc with stenosis	1 (4.0)	1 (11.1)	0
Compression fracture with stenosis	1 (4.0)	1 (11.1)	0
History of spine surgery (*n*, %)			
Yes	4 (16.0)	3 (33.3)	1 (6.3)
No	21 (84.0)	6 (66.7)	15 (93.8)
Osteoporosis (*n*, %)			
Yes	10 (40.0)	3 (33.3)	7 (43.7)
No	14 (56.0)	6 (66.7)	8 (50.0)
Missing	1 (4.0)	0	1 (6.3)
T score	−2.02 ± 1.17	−1.69 ± 1.28	−2.22 ± 1.10
BMD > −2.5	14 (56.0)	6 (66.7)	8 (50.0)
−3.0 < BMD ≤ −2.5	4 (16.0)	2 (22.2)	2 (12.5)
BMD ≤ −3.0	6 (24.0)	1 (11.1)	5 (31.3)
Missing	1 (4.0)	0	1 (6.3)
Number of OLIF surgery level			
1-level	18 (72.0)	7 (77.8)	11 (68.8)
2-level	5 (20.0)	1 (11.1)	4 (25.0)
3-level	2 (8.0)	1 (11.1)	1 (6.3)
Surgery level (*n*, %)			
L1-2	1 (4.0)	1 (11.1)	0
L2-3	2 (8.0)	2 (22.2)	0
L3-4	2 (8.0)	1 (11.1)	0
L4-5	14 (56.0)	3 (33.3)	11 (68.8)
L3-4-5	4 (16.0)	1 (11.1)	3 (18.8)
L2-3-4-5	2 (8.0)	1 (11.1)	1 (6.3)
L2-3, L4,5	1 (4.0)	0	1 (6.3)
Posterior decompression (*n*, %)			
Yes	11 (44.0)	1 (11.1)	10 (62.5)
No	14 (56.0)	8 (88.9)	6 (37.5)

Value expressed as count (%) or mean ± standard deviation.

Abbreviation: DM, diabetes mellitus; BMD, bone marrow density; OLIF, oblique lateral interbody fusion; L, lumbar.

The peri-operative characteristics and complications are shown in Table 2. Patients who underwent the two-stage surgery compared with the one-stage procedure had longer operation times (355.4 vs. 196.6 min), and longer hospital stays (6.5 vs. 4.7 days). In addition, the two-stage surgery was associated with a greater likelihood of having a blood transfusion compared with the one-stage surgery (12.5% vs. 0%). Following OLIF surgery, 96% of patients were without subsidence 3 months after surgery. At 6 months, 80% of patients were without subsidence, and 1 year after surgery this was reduced to 72% (Table 3). 96% of patients achieved fusion at 1-year follow-up. At 1-year after OLIF treatment, there was a higher proportion of grade I subsidence (25% vs. 11.1%) and grade II fusion (43.83% vs. 22.2%) in the two-stage OLIF, compared with the one-stage OLIF.

**Table 2:** Perioperative parameters and complications.

	Total, *n* = 25	One-stage, *n* = 9	Two-stage, *n* = 16
Blood loss (ml)			
Minimal	12 (48.0)	5 (55.6)	7 (43.8)
50–150	9 (36.0)	3 (33.3)	6 (37.5)
>150	4 (16.0)	1 (11.1)	3 (18.8)
Blood transfusion	2 (8.0)	0	2 (12.5)
Operation time (min)	285.4 ± 122.4	196.6 ± 87.4	335.4 ± 111.7
1-level	239.4 ± 87.0	173.6 ± 78.0	281.4 ± 65.2
2-level	400.2 ± 144.9	214	446.8 ± 116.4
3-level	412.5 ± 102.5	340	485
Length of hospital stay (day)	5.8 ± 3.2	4.7 ± 2.5	6.5 ± 3.4
Peri-operative complication			
Endplate damage	0	0	0
Segmental artery injury	0	0	0
Bed-rest time (h)^a^	16.2 ± 3.5	18.3 ± 3.4	15.0 ± 3.1

Value expressed as count (%) or mean ± standard deviation.

^a^ For early mobilization, patients were encouraged to walk on post-operative day-1. Therefore, the longer the operation time, the bed rest time would appear shorter, relatively.

**Table 3:** Subsidence and fusion rate.

	Total, *n* = 25	One-stage, *n* = 9	Two-stage, *n* = 16
Subsidence grade (3 months)			
No Subsidence	24 (96.0)	9 (100.0)	15 (93.7)
Grade I	1 (4.0)	0	1 (6.3)
Grade II	0	0	0
Grade III	0	0	0
Subsidence grade (6 months)			
No Subsidence	20 (80.0)	8 (88.9)	12 (75.0)
Grade I	4 (16.0)	0	4 (25.0)
Grade II	0	0	0
Grade III	1 (4.0)	1 (11.1)	0
Subsidence grade (1 year)			
No Subsidence	18 (72.0)	7 (77.8)	11 (68.8)
Grade I	5 (20.0)	1 (11.1)	4 (25.0)
Grade II	1 (4.0)	0	1 (6.3)
Grade III	1 (4.0)	1 (11.1)	0
Fusion grade (1 year)			
No Fusion	1 (4.0)	1 (11.1)	0
Grade I	15 (60.0)	6 (66.7)	9 (56.3)
Grade II	9 (36.0)	2 (22.2)	7 (43.8)

Post-operative complications are shown in Table 4. Postoperative ileus (40%), cage subsidence (28%), and dystonia (12%) were the most common in both groups. In the two-stage OLIF group, 43.8% of patients had postoperative ileus, whereas, in the one-stage OLIF group, 33.3% of patients had postoperative ileus. Clinical characteristics at baseline for patients with and without fusion 1 year after OLIF surgery are summarized in Table 5. A total of 8 patients had fusion and only one patient was with no fusion 1 year after one-stage OLIF surgery.

**Table 4:** Post-operative complications.

	Total, *n* = 25	One-stage, *n* = 9	Two-stage, *n* = 16
Transient leg weakness	0	0	0
Transient sensory change	1 (4.0)	0	1 (6.3)
Postoperatve ileus	10 (40.0)	3 (33.3)	7 (43.8)
Revision surgery	0	0	0
Wound infection	0	0	0
Dystonia	3 (12.0)	0	3 (18.8)
Cage subsidence	7 (28.0)	2 (22.2)	5 (31.3)

All symptoms were completely resolved at postoperative-3, 6-month follow-up.

**Table 5:** Summary of one-stage-OLIF patients.

	Total, *n* = 9	Fusion, *n* = 8	No fusion, *n* = 1
Age (year)	67.8 ± 9.8	65.9 ± 8.5	83.0 ± 0
Sex			
Male	2 (22.2)	2 (25.0)	0
Female	7 (77.8)	6 (75.0)	1 (100.0)
Comorbidities			
Hypertension	3 (33.3)	3 (37.5)	0
Hyperlipidemia	4 (44.4)	4 (50.0)	0
DM	3 (33.3)	2 (25.0)	1 (100.0)
Chronic renal disease	1 (11.1)	1 (12.5)	0
Diagnosis (*n*, %)			
Stenosis	0	0	0
Spondylolisthesis	7 (77.8)	7 (87.5)	0
Degenerative disc disease	0	0	0
Rupture disc with stenosis	1 (11.1)	0	1 (100.0)
Compression fracture with stenosis	1 (11.1)	1 (12.5)	0
History of spine surgery (*n*, %)			
Yes	3 (33.3)	2 (25.0)	1 (100.0)
No	6 (66.7)	6 (75.0)	0
Osteoporosis (*n*, %)			
Yes	3 (33.3)	3 (37.5)	0
No	6 (66.7)	5 (62.5)	1 (100.0)
T score	−1.69 ± 1.28	−1.61 ± 1.34	−2.3 ± 0
BMD > −2.5	6 (66.7)	5 (62.5)	1 (100.0)
−3.0 < BMD ≤ −2.5	2 (22.2)	2 (25.0)	0
BMD ≤ −3.0	1 (11.1)	1 (12.5)	0
Number of OLIF surgery level			
1-level	7 (77.8)	6 (75.0)	1 (100.0)
2-level	1 (11.1)	1 (12.5)	0
3-level	1 (11.1)	1 (12.5)	0
Surgery level (*n*, %)			
L1-2	1 (11.1)	1 (12.5)	0
L2-3	2 (22.2)	1 (12.5)	1 (100.0)
L3-4	1 (11.1)	1 (12.5)	0
L4-5	3 (33.3)	3 (37.5)	0
L3-4-5	1 (11.1)	1 (12.5)	0
L2-3-4-5	1 (11.1)	1 (12.5)	0
Perioperative complications	0	0	0
Posterior decompression (*n*, %)			
Yes	1 (11.1)	0	1 (100.0)
No	8 (88.9)	8 (100.0)	0
Hernia	0	0	0
Subsidence	2 (22.2)	2 (25.0)	0

Value expressed as count (%) or mean ± standard deviation.

Abbreviation: DM, diabetes mellitus; BMD, bone marrow density; OLIF, oblique lateral interbody fusion; L, lumbar.

**Figure 1:**
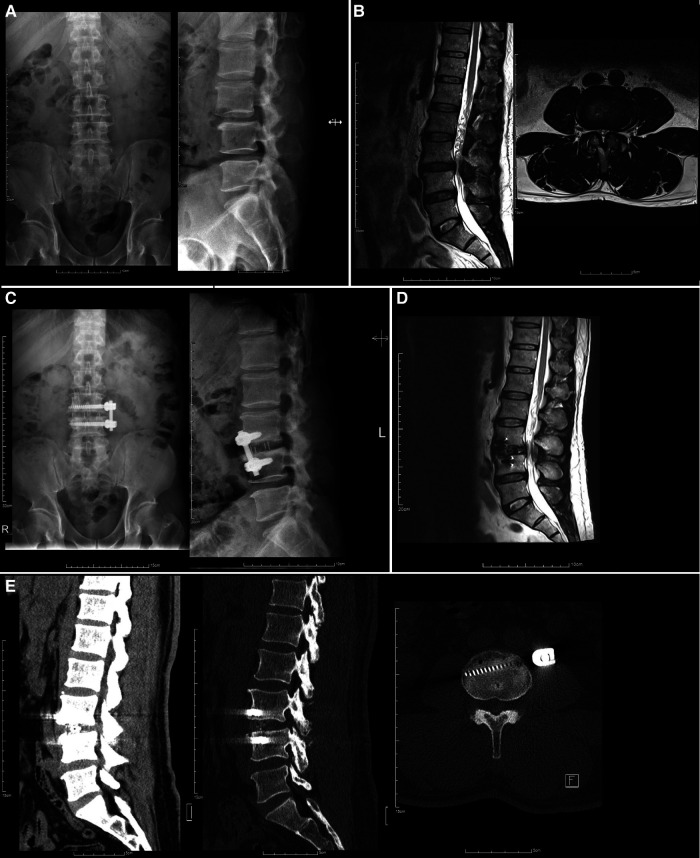
Preoperative and postoperative images of a 52-year-old man with L3-4 spondylolisthesis grade I who underwent one-stage OLIF. (**A**) Preoperative radiographs. (**B**) Preoperative MRI, (**C**) one-month postoperative radiographs, (**D**) 2-week postoperative MRI and (**E**) 5-month follow-up CT scan.

**Figure 2:**
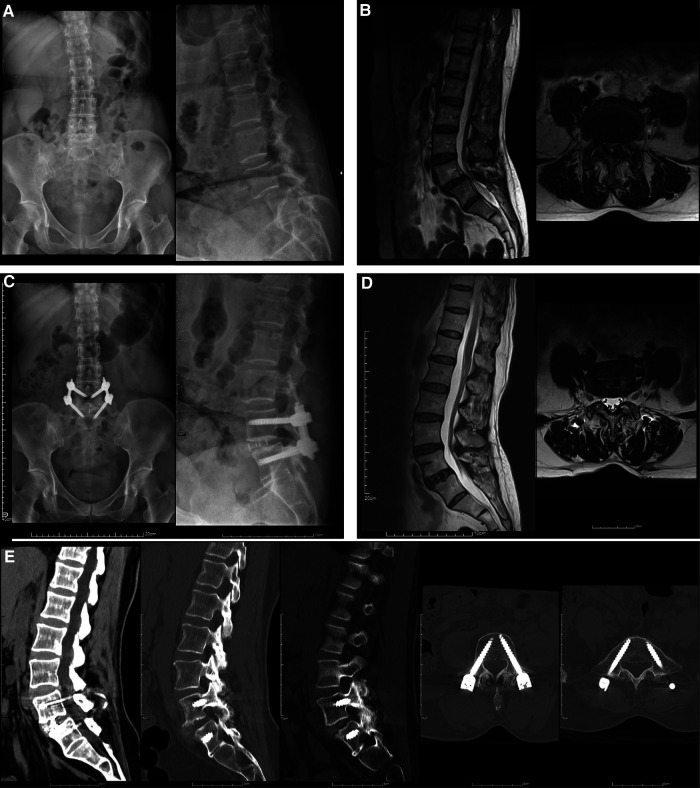
Preoperative and postoperative images of a 55-year-old woman with L4-5 spondylolisthesis grade II who underwent two-stage OLIF. (**A**) Preoperative radiographs. (**B**) Preoperative and MRI. (**C**) One-month postoperative radiographs. (**D**) 15-Month postoperative MRI and (**E**) 12-Month follow-up CT scan showed ankylosis of bilateral facet joint.

Exemplary preoperative and postoperative images obtained from patients who underwent one-stage OLIF or two-stage OLIF are shown in Figures 1, 2, respectively. Follow-up radiographic outcomes after OLIF are summarized in Table 6. Among all patients, anterior disc height (ADH), posterior disc height (PDH), and foraminal height (FH) significantly improved 1-week after OLIF, while retrolisthesis index (RI) and lumbar lordosis (LL) were significantly decreased. At the 1-year follow-up, decreased PDH and FH, and increased RI and LL were observed compared to the 1-week post-OLIF. For patients who underwent the one-stage OLIF, ADH (1.52 vs. 0.87 mm) and PDH (0.79 vs. 0.31 mm) significantly increased at 1-week after OLIF compared to baseline values. PDH (0.56 vs. 0.79 mm) and FW (0.96 vs. 1.03 mm) significantly decreased after 3 months compared to those values collected 1-week after OLIF. FH (1.79 vs. 2.09 mm) was significantly decreased after 6 months compared to 1-week after surgery. LL (42.89 vs. 34.57) was significantly increased after 6 months compared to 1 week after surgery. For patients who underwent two-stage OLIF, ADH (1.58 vs. 1.13 mm), PDH (0.92 vs. 0.40 mm), and FH (2.09 vs. 1.56 mm) significantly increased 1 week after OLIF compared to baseline values. RI (6.54 vs. 15.01 mm) and LL (39.28 vs. 43.41 mm) were significantly decreased 1-week after OLIF compared to baseline. In addition, PDH was significantly decreased at 3 months and 1 year after OLIF compared to 1 week after the surgery (0.76, 0.67 and 0.68 vs. 0.92 mm). LL was significantly increased 3 months and 6 months after OLIF compared to 1 week after surgery (44.23 and 47.20 vs. 39.28). The differences between radiographic parameters were not statistically different between one-stage and two-stage OLIF.

**Table 6:** Radiologic outcome.

	Total, *n* = 25	One-stage, *n* = 9	Two-stage, *n* = 16
**ADH (mm)**			
Pre-op	1.04 ± 0.33	0.87 ± 0.37	1.13 ± 0.28
Post-op (1 week)	1.56 ± 0.29^a,^*	1.52 ± 0.26^a,^*	1.58 ± 0.32^a,^*
Post-op (3 months)	1.55 ± 0.32	1.42 ± 0.30	1.62 ± 0.32
Post-op (6 months)	1.59 ± 0.25	1.55 ± 0.30	1.62 ± 0.22
Post-op (1 year)	1.60 ± 0.19	1.55 ± 0.14	1.63 ± 0.22
Difference^c^	0.12 ± 0.22	0.10 ± 0.24	0.13 ± 0.22
**PDH (mm)**			
Pre-op	0.37 ± 0.24	0.31 ± 0.19	0.40 ± 0.27
Post-op (1 week)	0.87 ± 0.29^a,^*	0.79 ± 0.38^a,^*	0.92 ± 0.22^a,^*
Post-op (3 months)	0.69 ± 0.34^b,^*	0.56 ± 0.27^b,^*	0.76 ± 0.35^b,^*
Post-op (6 months)	0.66 ± 0.24^b,^*	0.64 ± 0.30	0.67 ± 0.21^b,^*
Post-op (1 year)	0.65 ± 0.24^b,^*	0.61 ± 0.27	0.68 ± 0.23^b,^*
Difference^c^	−0.24 ± 0.26	−0.15 ± 0.29	−0.23 ± 0.26
**FH (mm)**			
Pre-op	1.67 ± 0.47	1.88 ± 0.46	1.56 ± 0.45
Post-op (1 week)	2.09 ± 0.40^a,^*	2.09 ± 0.28	2.09 ± 0.46^a,^*
Post-op (3 months)	1.95 ± 0.30^b,^*	1.86 ± 0.15	2.00 ± 0.35
Post-op (6 months)	1.93 ± 0.25	1.79 ± 0.19^b,^*	2.02 ± 0.26
Post-op (1 year)	1.88 ± 0.31^b,^*	1.82 ± 0.28	1.91 ± 0.34
Difference^c^	−0.14 ± 0.42	−0.22 ± 0.31	−0.10 ± 0.47
**FW (mm)**			
Pre-op	0.89 ± 0.31	0.79 ± 0.37	0.95 ± 0.26
Post-op (1 week)	1.01 ± 0.22	1.03 ± 0.21	0.99 ± 0.23
Post-op (3 months)	1.00 ± 0.29	0.96 ± 0.16^b,^*	1.02 ± 0.34
Post-op (6 months)	1.15 ± 0.29	1.03 ± 0.16	1.22 ± 0.33
Post-op (1 year)	1.16 ± 0.29	1.13 ± 0.24	1.17 ± 0.32
Difference^c^	0.13 ± 0.34	0.07 ± 0.16	0.16 ± 0.41
**Angle of L1-S1 (coronal)**			
Pre-op	3.21 ± 2.89	3.38 ± 2.69	3.12 ± 3.07
Post-op (1 week)	3.10 ± 2.98	3.15 ± 2.11	3.08 ± 3.45
Post-op (3 months)	2.81 ± 2.60	2.43 ± 1.93	3.00 ± 2.91
Post-op (6 months)	2.72 ± 2.71	1.99 ± 1.57	3.14 ± 3.18
Post-op (1 year)	3.15 ± 2.66	3.20 ± 2.22	3.12 ± 2.99
Difference^c^	−0.21 ± 3.01	−0.63 ± 2.61	0.03 ± 3.31
**Angle of disc level (coronal)**			
Pre-op	1.64 ± 1.97	1.90 ± 2.65	1.49 ± 1.54
Post-op (1 week)	1.33 ± 0.88	1.90 ± 1.04	1.00 ± 0.58
Post-op (3 months)	1.26 ± 0.98	1.76 ± 1.50	1.00 ± 0.49
Post-op (6 months)	1.44 ± 1.07	1.90 ± 1.38	1.17 ± 0.80
Post-op (1 year)	1.90 ± 1.51	2.18 ± 1.63	1.75 ± 1.49
Difference^c^	0.47 ± 1.77	−0.07 ± 1.91	0.79 ± 1.69
**Angle of disc level (sagittal)**			
Pre-op	7.30 ± 4.66	6.48 ± 4.37	7.77 ± 4.87
Post-op (1 week)	8.07 ± 4.60	7.90 ± 4.05	8.16 ± 5.01
Post-op (3 months)	9.71 ± 3.63	9.54 ± 4.45	9.79 ± 3.30
Post-op (6 months)	10.19 ± 5.82	9.87 ± 7.10	10.40 ± 5.14
Post-op (1 year)	9.77 ± 4.14	9.15 ± 5.08	10.10 ± 3.72
Difference^c^	1.78 ± 4.26	1.33 ± 4.46	2.03 ± 4.31
**RI (mm)**			
Pre-op	12.88 ± 8.31	9.09 ± 6.19	15.01 ± 8.76
Post-op (1 week)	5.35 ± 4.89^a,^*	3.23 ± 4.71^a,^*	6.54 ± 4.72^a,^*
Post-op (3 months)	7.12 ± 6.11	4.79 ± 5.05	8.29 ± 6.40
Post-op (6 months)	6.68 ± 5.65	4.97 ± 4.39	7.83 ± 6.26
Post-op (1 year)	8.48 ± 6.83^b,^*	6.18 ± 5.15	9.72 ± 7.48
Difference^c^	2.65 ± 4.33	2.03 ± 3.44	2.99 ± 4.83
**LL (angle)**			
Pre-op	41.53 ± 12.61	38.19 ± 14.73	43.41 ± 11.32
Post-op (1 week)	37.59 ± 13.51^a,^*	34.57 ± 16.87	39.28 ± 11.48^a,^*
Post-op (3 months)	44.83 ± 12.23^b,^*	46.03 ± 18.58	44.23 ± 8.19^b,^*
Post-op (6 months)	45.48 ± 13.79^b,^*	42.89 ± 19.42^b,^*	47.20 ± 8.95^b,^*
Post-op (1 year)	42.17 ± 14.82	45.51 ± 21.22	40.37 ± 10.62
Difference^c^	4.77 ± 11.93	8.60 ± 15.35	2.70 ± 9.71

Abbreviation: ADH, anterior disc height; PDH, posterior disc height; FH, foraminal height; FW, foraminal width; RI, retrolisthesis index; LL, lumbar lordosis.

^a^ Evaluated the difference between 1-week post-op and pre-op.

^b^ Evaluated the difference between each followed-up timepoint of post-op and 1-week post-op.

^c^ The difference between 1-year post-opand 1-week post-op.

* Statistically significant between pre-op and each time point of post-op by Wilcoxon sign rank test (*p* < 0.05).

## Discussion

It is generally accepted that anterolateral fixation provides satisfactory clinical outcomes and advantages, however, there is no clear guidance for choosing posterior or anterolateral fixation. In our present study, the clinical and radiographic efficacy of OLIF with anterolateral screw fixation alone (one-stage OLIF) and OLIF with conventional posterior percutaneous pedicle screw (two-stage OLIF) was evaluated in a group of patients with heterogenous lumbar diseases.

In our patients with heterogenous and multiple-level lumbar diseases, successful clinical outcomes were observed using the one-stage anterolateral surgical approach. Follow-up at 1 year after one-stage OLIF demonstrated radiographic evidence of satisfactory outcomes for all patients. One-stage OLIF was associated with shorter operation times and less blood loss, although the length of hospital stay and bed-rest time was similar to the two-stage OLIF group. Neither study group had peri-operative complications such as endplate damage or segmental artery injury. Less post-operative complications (e.g., cage subsidence, postoperative ileus, dystonia) were reported in patients who underwent one-stage OLIF compared to two-stage OLIF.

OLIF with supplement percutaneous pedicle screw fixation is widely used to improve fusion rates. Anterolateral screw fixation has been proposed to possibly attenuate the unwanted effects of OLIF with posterior pedicle screw fixation, such as increased operative time and the amount of intraoperative bleeding and fluoroscopy (21). In our study, screw fixation was performed with navigation assistance to improve accuracy and further reduce operative time, radiation exposure. Many factors contribute to the success of interbody fusion procedures. The OLIF-implanted cage is generally larger than those used in variations of this procedure, thus increasing the contact area with the endplate. It also plays a role in supporting the interbody axial pressure, stabilizing the anterior and central spinal columns, providing a good environment for interbody fusion, and improving the interbody fusion rate (22, 23). A recent study by Guo et al. reported lower fusion rates in OLIF with anterolateral screw fixation (AF) compared with posterior percutaneous screw fixation (PF) and suggested that PF may improve stability and interbody fusion. However, this result may have been due to an insufficient follow-up period to detect significant changes in fusion rates or the inability of anterior vertebral fixation to effectively avoid lumbar spinal lateral flexion and extension. Routine monitoring and avoidance of lateral flexion and extension activities within 3 months after surgery was recommended (18). In our present study, a high and satisfactory fusion rate was demonstrated in patients who underwent posterior (two-stage OLIF) (fusion rate 100%) and anterolateral (one-stage OLIF) (fusion rate 88.9%) screw fixation. Only one case of non-fusion occurred in the one-stage OLIF group at the one-year follow-up.

Improved outcomes following OLIF include intervertebral space height, intervertebral foramen area, and vertebral canal area (24, 25). However, little is known regarding the impact of the position of fixation on these improved outcomes. Sato et al. (2017) performed OLIF on 20 patients with lumbar degenerative diseases, demonstrating intervertebral space height, foramina height, and anteroposterior diameter of the thecal sac were significantly increased after surgery in two groups with different positions of fixation (OLIF + AF; OLIF + PF). However, no significant differences were reported between groups regardless of fixation position. These results suggest that OLIF + AF can maintain stability after lumbar fusion and achieve similar imaging outcomes as OLIF + PF (17). In our present study, 1 week after surgery the evaluated radiographic parameters ADH, PDH, FH, RI, and LL were significantly corrected. Over time there was a trend of gradual loss of correction for PDH, FH, RI, and LL; however, the observed loss of correction was similar between one-stage and two-stage OLIF groups, except for LL. The LL angle was larger in those patients who underwent posterior fixation, thus suggesting posterior fixation can maintain LL angle correction and minimize any loss of correction for a longer period.

In our study, both one-stage and two-stage OLIF groups and their associated fixation positions had a similar incidence of complications. Cage subsidence was observed in a total of 2 patients (22.2%) and 3 patients (31.3%) in the one-stage and two-stage OLIF groups, respectively. These results suggest that one-stage OLIF does not result in a greater incidence of subsidence. This is consistent with the radiographic findings, which also showed a similar loss of correction over time between groups, and imaging outcomes, which indicated similar maintenance of stability.

Fijubayashi et al. (2017) have reported the complication rate associated with OLIF was 15.3%, with the most common complications being sensory nerve injury and psoas major weakness, most of which gradually resolved (26). A multi-center retrospective study by Abe et al. (2017) reported an OLIF complication rate of 48.3%. Most of these complications were surgery-related and self-limiting, such as endplate injury collapse (18.7%), and temporary psoas major muscle weakness and thigh numbness (13.5%). The incidence of serious surgical complications of OLIF was 1.9% which included vascular, nerve, and ureteral injuries (27). No cases of peri-operative complications in either group were reported in our present study nor were peri-operative complications (e.g., endplate damage or segmental artery injury). The most common postoperative complication in our study was post-operative ileus and dystonia (for two-stage OLIF, but not one-stage OLIF). No postoperative residual low back pain was reported in our patients with posterior fixation, suggesting posterior incision of the paravertebral muscle for screw positioning did not result in significant paravertebral muscle injury or denervation (28). All complications resolved after treatment, suggesting the safety of both one-stage and two-stage OLIF for lumbar diseases.

Ankylosis of facet joint and facet joint degeneration is a well-known consequence of spinal fusion. When evaluating fusion, complete ankylosis of the zygoapophyseal joints also indicates true spinal fusion as defined by evaluation criteria e.g., Pathria classification system grade III. This is because the disc and the zygapophyseal constitute an articular complex, once the anterior fusion is achieved, posterior ankylosis is expected, thus, increased immobilization due to screws may determine higher chances for segmental fusion, in other words, anterior fusion or a solid fixation of the segment due to the implants may determine immobilization and zygapophyseal ankylosis (as discussed in the Proletti et al., 2020 study (29). A recent study investigating the risk factors affecting the incidence of facet joint degeneration found that fixation time is a risk factor. They found that the earlier bony union occurs, and internal fixation was removed, the incidence of facet joint degeneration was lowered (30). Further studies are required to clarify the effect of intersegmental pedicle screw fixation and to evaluate various risk factors that may predict the possibility of degeneration of the intervertebral disc and facet joints.

**Figure 3:**
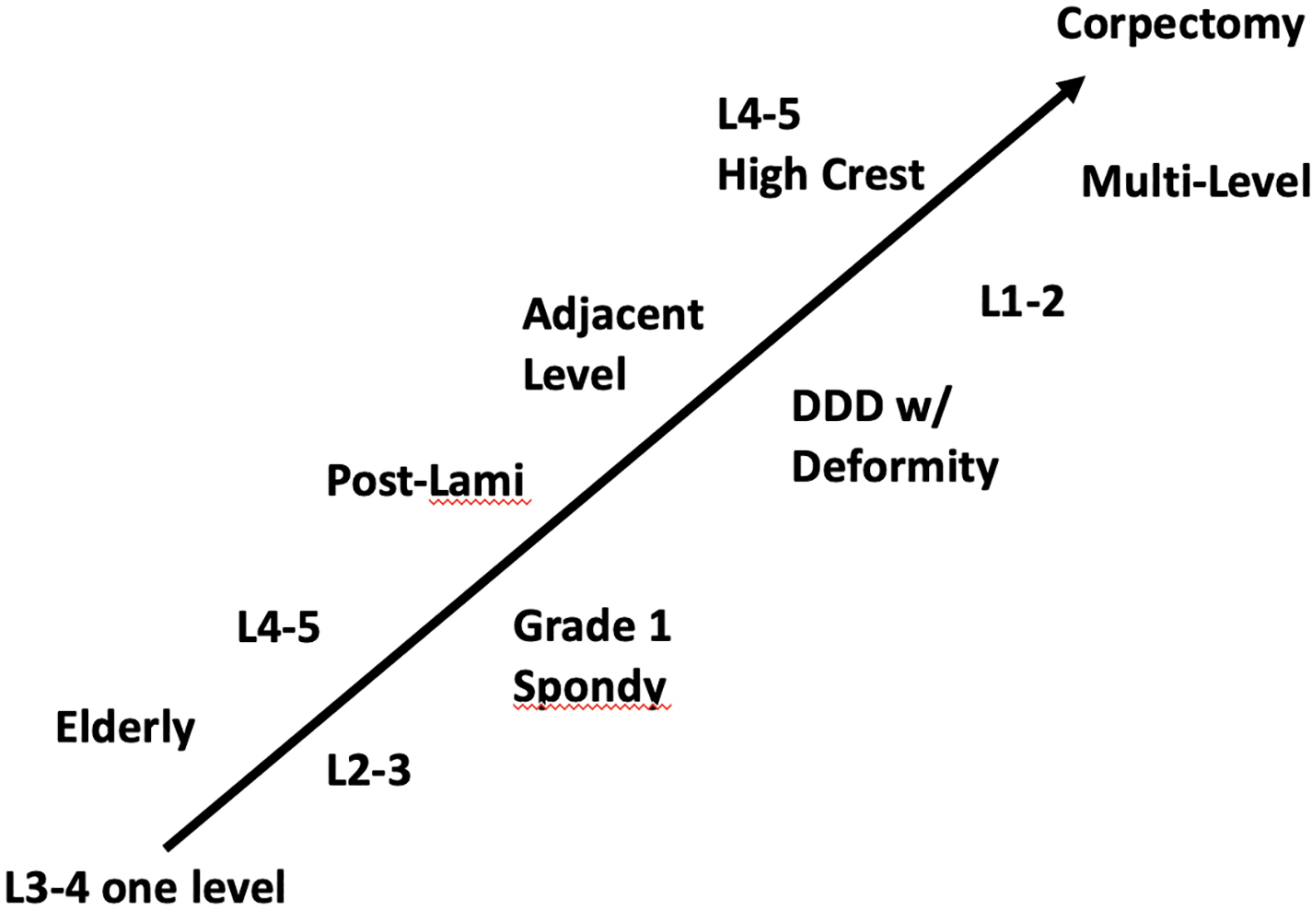
Cascade of surgery complexity.

Based on the current results, one-stage OLIF should be considered for patients with spondylolisthesis (≤Grade 1), herniated intervertebral disc (disc height < 1/2 requires fusion), or patients with multiple previous posterior operation who subsequently had adjacent segment degeneration). At this time, we can't make a recommendation for patients with osteoporosis to be potential candidates for one-stage OLIF. Considering the learning curve for performing OLIF, specifically how the lateral incision tends to be more difficult than expected, we suggest that for one-stage OLIF the initial series of cases should be less complex (e.g., single-level L3-4, L2-3, L4-5). With experience and skill, more complex cases are feasible (multi-level, L1-2) (Figure 3). In our case series, we demonstrated success with one-stage OLIF for patients requiring multi-level surgery, as well as the single level at L1-2. It should be noted that even though the OLIF approach provides safe access to nearly all lumbar levels because the wide interval between the psoas and aorta allows for a straightforward left-sided oblique approach to the discs above L5. However, for L5-S1 segments, modifications in the technique are required (31), and ALIF approach is the preferred approach currently for L5-S1 (32). Furthermore, because OLIF is performed from an oblique trajectory, one of the difficulties of this approach has been the decrease in direct visualization of the surrounding anatomy. Navigation could be applied as an alternative approach to fluoroscope, for guidance for localization and orientation (33, 34).

There are several strengths and limitations to the current study worth noting. The study was a single-center, retrospective study design, with a small sample size. Considering a preoperative VAS score was not available in the medical records, post-operative improvement in low back pain was not examined between one-stage and two-stage OLIF. However, because one-stage OLIF does not require paravertebral muscle dissection, it was assumed patients would have lower postoperative VAS scores. There were no complaints of pain from patients in either group noted during follow-up visits. This study included heterogenous lumbar diseases with surgery performed on a variety of segments of the spine. Different spinal segments may have varying levels of complexity and potential for complications. Our study demonstrated that patients with heterogenous lumbar disease can be suitable for one-stage OLIF. Clinical and radiographic outcomes were improved at one-year post-surgery; however, longer follow-up is required to ensure positive long-term clinical outcomes.

In conclusion, the current study demonstrated satisfactory clinical outcomes in the short term and over 1 year of follow-up in patients who underwent one-stage OLIF. This surgical approach could be considered for patients with spondylolisthesis (≤Grade 1), or patients with multiple previous posterior fixation who subsequently had adjacent segment degeneration. One-stage anterolateral fixation can also be considered in cases with greater operative complexity with an experienced surgeon. Longer clinical follow-up is necessary to confirm long-term outcomes.

## Data Availability

The original contributions presented in the study are included in the article/Supplementary Material, further inquiries can be directed to the corresponding author.
